# Rubber Material-Model Characterization for Coupled Thermo-Mechanical Vulcanization Foaming Processes

**DOI:** 10.3390/polym14061101

**Published:** 2022-03-09

**Authors:** Noelia Alcalá, Mariana Castrillón, Ismael Viejo, Salvador Izquierdo, Leticia A. Gracia

**Affiliations:** Instituto Tecnológico de Aragón, C/María de Luna 7-8, 50018 Zaragoza, Spain; mariana.castrillon@saica.com (M.C.); iviejo@itainnova.es (I.V.); sizquierdo@itainnova.es (S.I.); lgracia@itainnova.es (L.A.G.)

**Keywords:** kinetic reaction, vulcanization, foaming, coupling, material model

## Abstract

A novel experimental methodology is developed for the characterization of the vulcanization and foaming processes of an ethylene propylene diene (EPDM) cellular rubber and for establishing the relationship of its physical and mechanical property evolution with vulcanization and foaming process temperature. To establish this relationship, the vulcanization and foaming reaction kinetics and their coupling have been determined, as well as important parameters in the behaviour of the material, such as conductivity, specific heat capacity and coefficients of expansion and foaming. This aforementioned strategy allows the setting of a material model that can be implemented into finite-element (FE) codes to reproduce the material changes during the vulcanization and foaming processes. The material model developed reproduces with enough accuracy the coupling of chemical kinetics of vulcanization and foaming reactions. The results provided by the numerical material model fit a similar trend, and values with an accuracy of 90–99% to those observed in the experiments conducted for the determination of the cellular rubber expansion in function of the temperature. Moreover, the cellular rubber expansion values agree with the structural analysis of vulcanized and foamed samples at different isothermal temperatures and with the proportional loss of mechanical properties in the function of the vulcanization and foaming degree.

## 1. Introduction

### 1.1. Cellular Polymers

The first cellular polymers were made in the late 1920s, with the production of latex foam, reaching industrial stages in the 1930s with Dunlop and Talalay processes [1,2]. After that, the technology of polymeric foams was applied to polyurethane and synthetic rubbers. Presently, a large number of rubber-based foams are commercially available attending to their chemical structure: based on polyurethane (PU), ethylene vinyl acetate (EVA) copolymer, natural rubber (NR), styrene-butadiene rubber (SBR), chloroprene (CR), alkyl acrylate copolymer (ACM), ethylene propylene diene (EPDM) terpolymer and acrylonitrile butadiene rubber (NBR), silicon rubber and so on [2,3,4]. Cellular rubbers became very important in commercial applications due to their lower density and therefore weight reduction, thermal and acoustic insulation, energy absorption and for the possibility of producing shaped components to fit irregular spaces. Foam properties are generally governed by the number, size and morphology of cells and mechanical properties of the base polymer [3,5]. Due to the excellent properties of EPDM elastomers, such as good weather-resistance, high resistance to acid, alkali, oxygen and ozone, insulation properties, high tear, impact and abrasion resistance and high and low temperature performance [2,3,6], they have been increasingly used as cellular material since the beginning of the 1990s. For these reasons, EPDM rubbers are especially used for the automotive industry for hoses, wipers, bumpers, gaskets, pipe insulation, door seals and weather-stripping [2,6,7]. The excellent properties of the EPDM elastomers are related to their chemical structure. EPDM is a synthetic copolymer of ethylene and propylene with a diene as co-monomer, which provides crosslinking sites for vulcanization. The presence of propylene and the lack of unsaturated double bonds in the main chain of EPDM avoid the crystallinity and confer its stability properties [6,8]. The last letter M refers to the polymethylene (CH_2_) type of backbone according to the nomenclature given by ISO 1629. The average molecular weight of EPDM lays between 30,000 and 150,000 g/mol, depending on the polymerization and the ethylene/propylene/diene ratio.

There are two main methods for producing foamed materials in the industry: physical or chemical foaming. The physical foaming process normally consists of introducing a solvent or inert gas as a supercritical fluid into a thermoplastic melt under high pressures. Then, in the extrusion or injection moulding step, it evaporates when the pressure is reduced, and the polymer melt expands. In this process, a large amount of gas is produced, and the cell structure obtained depends on the extrudate viscosity achieved during the cooling step. Therefore, the stability and homogeneity of the foam structures are closely related to the process reproducibility. This fact limits the commercial usage of the physical foaming process due to it, requiring complex mixing equipment and a high-precision process control of the blend [8,9].

Alternatively to physical foaming processes, in the chemical-foaming ones, inorganic or organic blowing agents (referred to as BA from now onwards) are added to the rubber compounds during the mixing process. Their thermal decomposition reaction during the vulcanization process causes material expansion, due to the generation of gaseous products. Since the early 1950s, the use of chemical blowing agents, such as toluene sulphonyl hydrazide (TSH) and 4,4′-oxybis(benzene sulphonyl hydrazide) (OBSH) has become very usual in the chemical-foaming process [9]. Specifically, OBSH is a BA widely used in the production of EPDM cellular compounds.

### 1.2. Experimental Characterization

In chemical foaming, the thermal decomposition reaction of the BA takes place in the same range of temperatures of the vulcanization process. Therefore, it can be stated that both reactions occur in parallel [2,7]. Consequently, this kind of foaming mechanism also requires a high knowledge of decomposition and vulcanization kinetics since they can interact with each other and modify the final cellular structure [2,7,9]. For producing stable and uniform foam structures with a good reproducibility, it is essential to obtain well-distributed BAs on the polymer matrix and to control the processing parameters as much as possible. Both the temperature and the time in foaming and vulcanization kinetics have a large influence on the most important material process parameters, such as, for instance, the rubber compound viscosity. If the blowing reaction is produced too early, the gas can expand without restriction, producing extremely large bubbles that even can collapse [2,9]. However, if the vulcanization process is produced before the decomposition reaction of the BA, gas expansion is limited and as a result, the bubbles grow too small, and the foaming mechanism becomes inefficient. Hence, to have a proper generation of elastomeric foams, it is important to know the process temperatures precisely. Moreover, vulcanization and foaming processes are influenced by the composition of the rubber with a blowing agent (“rubber with blowing agent” is referred as rubber+BA from now onwards) [2,7,8,9]. Therefore, the production of cellular rubbers requires a high level of knowledge to avoid quality fluctuations in the final product.

**Figure 1:**
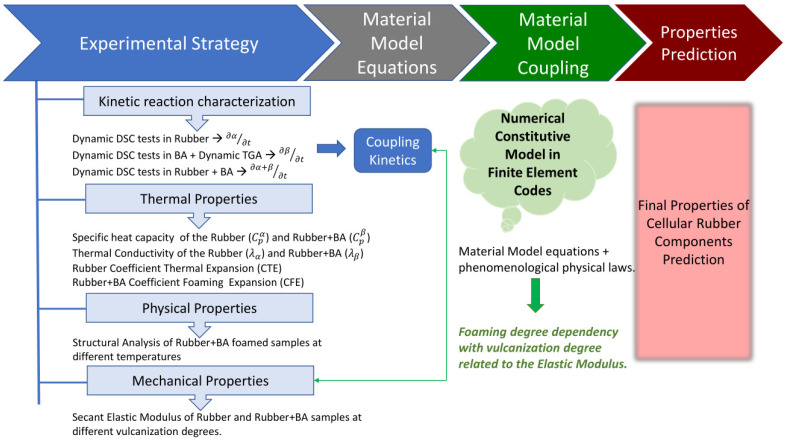
Schematic diagram depicting the different steps of the experimental strategy followed to calibrate a numerical model with the ability to predict coupled thermo-mechanical-vulcanization-foaming processes.

The experimental strategy designed in the present paper attempts to reproduce the coupling of the vulcanization and foaming kinetic reactions to optimize manufacturing conditions and to determine their relationship with the evolution of the physical and mechanical material properties of the final rubber material. In order to obtain the necessary parameters to model the rubber transformations during the process conditions, first dynamic Differential Scanning Calorimetry (DSC) tests, which measure the heat flow in the sample to maintain the heating rate set in the experiment, are performed in the EPDM matrix (referred to as rubber from now onwards), in the BA, and in the rubber+BA. DSC tests allow the obtaining of their kinetics and establishment of the interaction of both vulcanization and foaming kinetic reactions when they are produced simultaneously. After the decomposition reaction of the BA, a residue remains in the pan and the effective BA mass decomposed during the foaming reaction can be measured by means of a thermogravimetric analyzer (TGA). Second, rubber thermal properties are then determined to define a heat transfer equation. In particular, specific heat capacity ($C_{p}$), conductivity (λ) and thermal expansion (CTE) for the rubber and foaming expansion coefficient (CFE) for the rubber+BA are obtained for that purpose. The $C_{p}$ and λ of the rubber are obtained by a set of specific tests designed in the DSC device using some reference materials. The same parameters for the rubber+BA are calculated by means of a mixing rule that considers $C_{p}$ and λ of the rubber and the air trapped in the bubbles, which are produced inside the rubber. For determining the CTE during vulcanization and the CFE of the rubber+BA, an experimental set-up is developed in a laboratory rheometer. Finally, the porosity level, the cell diameter at different reaction temperatures, and the mechanical properties in the function of the vulcanization degree (curing) are also determined. The porosity in terms of cell area is measured experimentally from portions of rubber+BA, vulcanized and foamed in the rheometer at different temperatures, by using a stereoscope and an image analysis software. The mechanical property evolution in rubber and in rubber+BA are determined in tensile mode from samples prepared by die-cutting from rubber discs, previously vulcanized in the laboratory rheometer at one isothermal temperature and at certain vulcanization and foaming degrees. The aforementioned experimental strategy is depicted in Figure 1 for a better understanding.

### 1.3. Constitutive Model

The experimental characterization explained in Section 1.2 allows the establishment of material-model equations to determine not only the vulcanization and foaming behaviour, but also their chemical interaction and relationships with the evolution of other rubber properties. To reproduce the material property interactions, a numerical constitutive model is defined for its implementation in finite-element (FE) codes by means of the application of the material-model equations and phenomenological physical laws to calibrate models based on experimental results. The experimental methodology conceived to determine the CFE is used to calibrate the bubble growth approach, which is defined in terms of displacement instead of in terms of other traditional mechanisms based on viscosity, surface tension [10,11,12,13] or pressure inside the bubble [13].

### 1.4. Objective

Several authors have studied phenomena produced in the foaming process from different points of view, such as, for example, the study of the vulcanization and foaming kinetics in foamed rubber compounds [2,14], the effect of foaming temperature on average cell size [3,4,5,7], on crosslink density [3,5] and on mechanical properties [3,4,5], or even the effect of hydrostatic pressure in the thermal conductivity properties and in the porosity of the foamed rubber [15]. However, the present study investigates the foaming process in more detail. It does not only study the influence of different variables in the final foamed rubber product, but also establishes relationships among the coupling of chemical reactions (vulcanization and foaming kinetics), thermal, mechanical and physical properties evolution of the rubber+BA in the function of exposure times and vulcanization process temperature. Therefore, the novel experimental methodology allows the determination of the evolution of the material properties during the manufacturing process of this kind of cellular material and their interaction according to vulcanization process parameters from an extent point of view. Hence, the experimental methodology developed allows the obtaining of sufficient parameters to set constitutive equations and provides information to implement a numerical material model to predict the final properties of components made of foamed rubber compounds.

The numerical material model presented in the paper focuses on reproducing a real-time automotive door seal extrusion line in which rubber+BA is involved. The resulting data obtained from the FE simulation model are extremely valuable to increase the knowledge on the evolution of the rubber properties during the manufacturing process. The selection of the processing conditions impacts directly in the evolution of the kinetics reaction and in the physical and mechanical changes that the elastomer suffers along the extrusion line. Consequently, it is essential to enhance their knowledge to be able to achieve the optimal properties and shape of the final profile, which normally is fitted by trial-and-error process and which depends on molecular changes happening during the vulcanization process.

## 2. Materials

The material under study consists of a rubber matrix of EPDM mixed with OBSH chemical BA that begins to decompose in the same range of temperature at which vulcanization reactions start, i.e., both chemical reactions develop themselves in parallel [2,14]. The decomposition temperature of the OBSH is around 140–160 °C, creating closed-cell structures in the rubber compound [2,9,14]. Material samples are supplied by the company Standard Profil as strips of unvulcanized rubber blend. To obtain sheets, from which testing samples can be cut, a portion of these strips are cut and processed in a roll mill. The composition of the EPDM blend is undisclosed by the company. The vulcanization system is based on sulphur and the EPDM compound contains between 1 % and 3 % BA.

## 3. Methods and Results

This section details the experimental strategy designed to obtain results for modelling the parameters involved in the vulcanization and foaming processes of the rubber+BA used in this study.

### 3.1. Vulcanization Reaction

**Figure 2:**
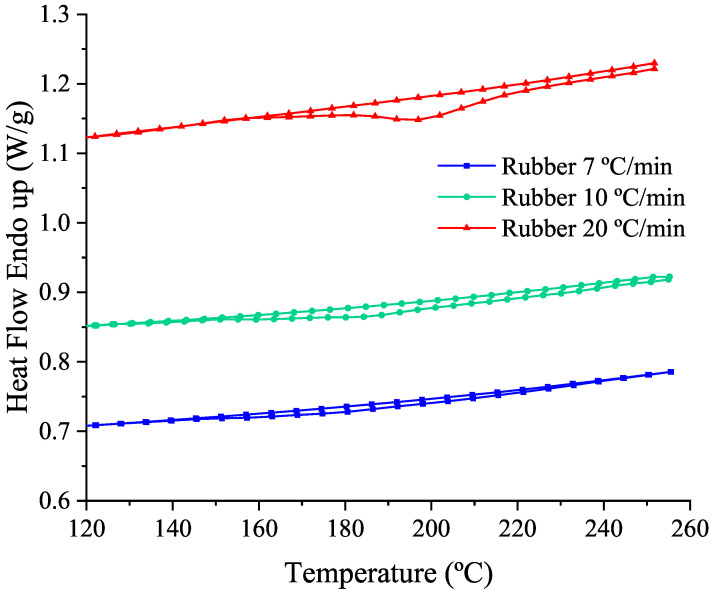
Thermograms from dynamic DSC tests for the rubber sample at 7, 10 and 20 °C/min heating rates.

DSC measurements at a constant temperature rate are carried out to obtain the energy necessary to achieve a complete vulcanization reaction and the vulcanization degree of dependence with time. These dynamic DSC tests are performed according to ISO 11357-1 recommendations with a Perkin Elmer DSC6 device. A nitrogen flow rate of about 50 mL/min passes through the cell and an empty pan is used as the reference material. The mass of the sample is between 35 and 40 mg. Three different heating rates are used for the dynamic measurements: 7, 10 and 20 °C/min and the temperature is controlled in the range from 10 °C to 260 °C. Figure 2 shows that as the heating rate increases the vulcanization temperature peak moves to higher temperatures from 183.29 °C at 7 °C/min to 197.25 °C at 20 °C/min. The exothermic enthalpy related to the vulcanization is obtained from the area enclosed between the first and the second heating step (once the rubber is completely vulcanized) of the DSC thermogram represented in function of time instead of temperature. Thus, the vulcanization enthalpy obtained is between 4–5 J/g.

The vulcanization mechanism of an EPDM rubber consists of a series of parallel and sequential reactions, and, by assuming that the heat generated during the process is only due to the crosslinking reaction, the rubber vulcanization degree and vulcanization kinetic can be determined by means of a DSC test. This technique is especially appropriate due to its high sensitivity in heat determination and the small sample size required [16]. Curing rubber state can be obtained as the ratio of the instantaneous heat and the total heat of the reaction [2,14] in an equivalent way to that described in the standard ISO 11357-5. The heat generation associated with the vulcanization degree, α, that indicates the degree (from 0 to 1) of completion of the vulcanization reaction, is then modeled according to Equation (1):

(1) $$\dot{Q}=Q_{total}^{\alpha }\frac{\partial \alpha }{\partial t}$$

where $Q_{total}^{\alpha }$ is the total heat of the reaction associated with the rubber, in W/m^3^ and ∂α/∂t is the vulcanization kinetic.

The vulcanization kinetic is commonly defined as a Kamal–Sourour semi-empirical auto-catalytic reaction model [17,18]. In this work, this model is modified as follows:

(2) $$\frac{\partial \alpha }{\partial t}=\left(K_{1\alpha }+K_{2\alpha }\alpha^{m}\right){\left(B_{\alpha }-\alpha \right)}^{{}^{n}}$$

where α is the vulcanization degree (dimensionless), *m* and *n* are the reaction orders, $B_{\alpha }$ is the additional parameter to lock the maximum value of α and *K* coefficients are defined in Arrhenius-type temperature dependence:

(3) $$K=Ae^{-E/RT}$$

where *E* is the activation energy in J × mol^−1^, A is the pre-exponential factor, R the universal gas constant in J × mol^−1^ × K^−1^ and *T* is the absolute temperature in Kelvin.

### 3.2. Foaming and Foaming Evolution Reaction

Dynamic DSC tests are performed on BA material to determine the decomposition reaction as a function of temperature [2,14] with independence of its combination with rubber. For this purpose, a power compensation Perkin Elmer DSC Diamond instrument is used. The BA reaction is extremely fast, and a power compensation DSC provides better resolution for sharp events. The DSC is operated with a nitrogen flow rate of about 50 mL/min through the cell and an empty pan is used as the reference material. The mass of the sample is between 5 and 6 mg and the temperature change is controlled from 10 °C to 220 °C at 2.5, 5, 7 and 10 °C/min.

**Figure 3:**
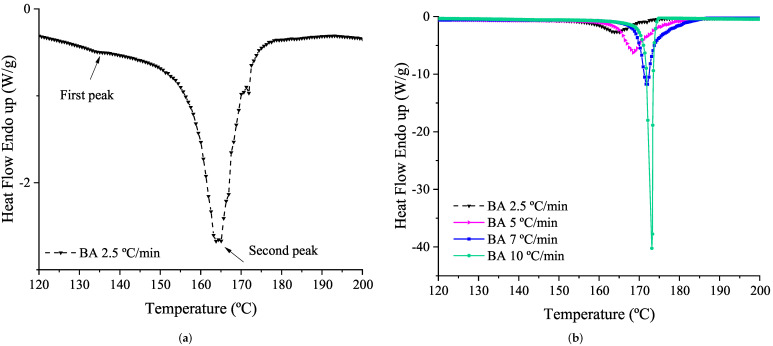
Blowing Agent (BA) decomposition exothermic peak from dynamic DSC tests at 2.5 °C/min (**a**) and 2.5, 5, 7 and 10 °C/min heating rates (**b**). Please note that the curve in Figure 3a is the same as the black one in Figure 3b, which is shown isolated for a better identification of the exothermic peaks.

Dynamic DSC results in Figure 3 show two decomposition peaks of the BA. The first one, much smaller, appears at around 135–150 °C and generates an insignificant enthalpy energy of 5–6 J/g. The second one, which begins to arise from 150 °C at the minimum heating rate of 2.5 °C/min, reaches its maximum decomposition reaction rate at 163.44 °C. The peak temperature at the different heating rates of this second decomposition is included in Table 1. The second exothermic peak, at higher heating rates, is sharper and appears in a narrower range of the reaction duration, i.e., between the beginning and the end of the reaction there is only 10–20 °C, which would suggests that the reaction is almost explosive. As the heating rate increases, the exothermic reaction takes place at higher temperatures. Please note that at 10 °C/min the DSC device is not able to record accurately the variation in the heat flow (see Figure 3b, green line). This fact can be recognized in the lower enthalpy value (432.96 J/g) obtained at 10 °C/min (see Table 1) compared with the enthalpy value obtained at 7 °C/min. The enthalpy associated with the exothermic decomposition reaction of the BA corresponds to the area enclosed by the peak curve of the DSC thermogram represented in function of time instead of temperature, which includes the total energy of both peaks. Therefore, the enthalpy of decomposition reaction selected corresponds with the maximum value obtained of 511.93 J/g (being 5.37 J/g the enthalpy of the first peak and 506.56 J/g the one of the second peak).

The exothermic foaming process and the heat generation rate to be included in the numerical model is defined by Equation (4):

(4) $$\dot{Q}=Q_{total}^{\beta }\frac{\partial \beta }{\partial t}$$

where $Q_{total}^{\beta }$ is the total heat of the reaction associated with the foaming agent or BA, expressed in W/m^3^ and ∂β/∂t is the foaming kinetic, β being the foaming degree ranging from 0 to 1, when the foaming reaction is complete.

The foaming reaction can be, as in the case of the vulcanization reaction, defined by the Kamal–Sourour model:

(5) $$\frac{\partial \beta }{\partial t}=\left(K_{1\beta }+K_{2\beta }\beta^{m}\right){\left(B_{\beta }-\beta \right)}^{n}$$

where β is the foaming degree (dimensionless), *m* and *n* are the reaction orders, $B_{\beta }$ is the additional parameter to lock the maximum β reachable and Ks are defined in Arrhenius-type temperature dependence in the form of Equation (2).

**Figure 4:**
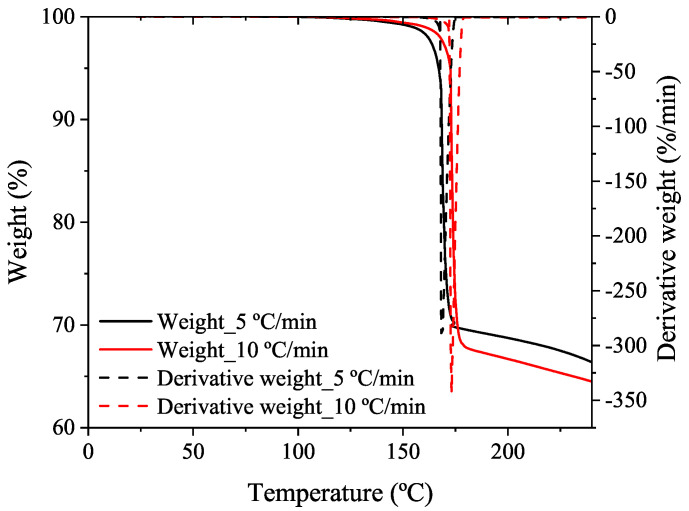
First weight loss of the BA (measured in the TGA dynamic analysis at 5 and 10 °C/min heating rates in nitrogen atmosphere) associated with the decomposition reaction of BA.

After the decomposition reaction of the BA, a residue remains in the pan. Consequently, the change in mass during the decomposition reaction is measured using a Perkin Elmer thermogravimetric analyzer (TGA). This device detects the mass loss with a resolution of 0.1 μg as a function of the temperature. The samples are evenly and loosely distributed in an open sample pan with an initial weight of 10–11 mg. The BA powder is heated at two different heating rates of 5 °C/min and 10 °C/min from 30 °C to 600 °C in a nitrogen atmosphere, which passes continuously through into the furnace at a flow rate of 50 mL/min. Figure 4 presents the thermogravimetric decomposition process of the BA. The decomposition reaction of the BA occurs abruptly in the range from 150 °C to 190 °C, where the slope of the weight curve resulting from the TGA experiment, expressed as the weight loss in percentage regarding the initial BA weight introduced in the pan, is more pronounced. Approximately only the 30–33% of the BA material decomposes and almost of 67–69% of the initial weight remains in the pan as residue. The maximum decomposition reaction takes place at 168.98 °C and 173.16 °C at 5 °C/min and 10 °C/min, respectively, according the derivative weight curve. The peak temperatures are extremely close to those obtained in the DSC tests performed in BA samples at both heating rates, despite using two different measuring devices. This fact shows the robustness and reproducibility of the BA decomposition results.

### 3.3. Rubber Coupled Vulcanization-Foaming Kinetics

Dynamic DSC tests are performed in the rubber+BA to determine and confirm whether the BA decomposition reaction and/or rubber curing is affected by the presence of one or both [2,4,14].

**Figure 5:**
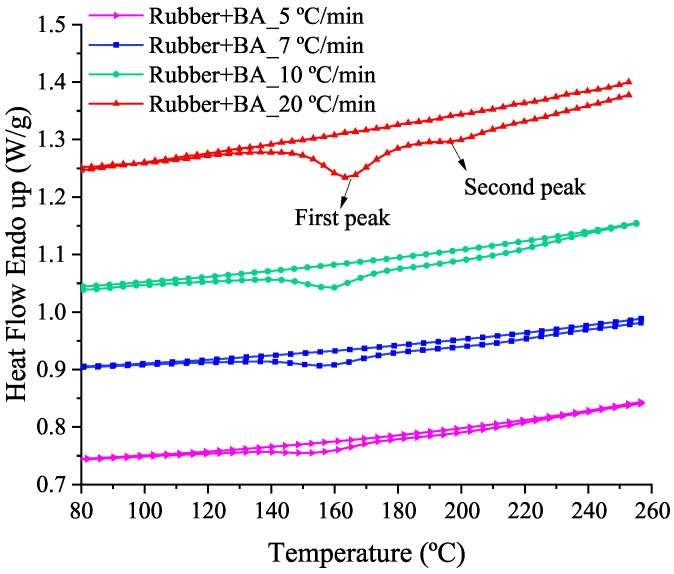
Thermograms from the dynamic DSC tests of the rubber+BA at 5, 7, 10 and 20 °C/min heating rates.

DSC tests are conducted in a Perkin Elmer DSC6 instrument using empty pans for the reference material and rubber+BA samples following the same procedure as already explained for the rubber (see Section 3.1). The mass of the sample is between 35 and 40 mg. Four different heating rates are used for the dynamic measurements: 5, 7, 10 and 20 °C/min. The temperature change is controlled from 10 °C to 260 °C. A high-purity nitrogen stream passes continuously through into the furnace at a flow rate of 50 mL/min. DSC thermogram curves resulting from these measurements are depicted in Figure 5. Table 2 shows numerical values for enthalpy and predominant peak temperature (T peak) for the rubber+BA sample at the different heating rates, but a second peak can also be appreciated at higher temperatures; both peaks are tagged in Figure 5 in a DSC curve at 20 °C/min but they appear at the different heating rates tested. Attending to the T peak values, the foaming-vulcanization reaction moves to lower temperatures with respect to the vulcanization and foaming reactions separately (Figure 2 and Figure 3, respectively).

**Figure 6:**
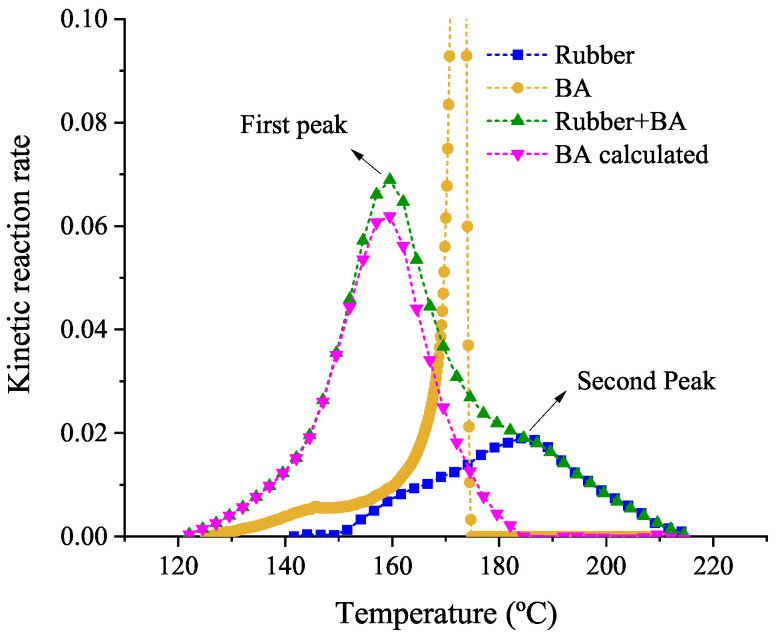
Comparison of experimental kinetic reaction rate evolution of rubber, rubber+BA and BA samples obtained from dynamic DSC results at 10 °C/min, and BA kinetic reaction rate curve calculated from experimental rubber+BA and rubber kinetic reactions.

The DSC results for the vulcanization and foaming reactions and for the coupled one are compared in terms of the kinetic reaction rate. The comparison for the case of 10 °C/min is shown in Figure 6, being similar for the rest of conditions. The kinetic reaction rate is defined as the vulcanization or foaming degree rate scaled by the enthalpy of the reaction and the mass fraction of each material that are included in the elastomer compound. For the specific case of the rubber+BA, the experimental coupled kinetic reaction rate (green curve in Figure 6) should result from Equation (6):

(6) $$\Delta H^{\alpha +\beta }\times \frac{\partial (\alpha +\beta )}{\partial t}=wt_{Rubber}\times \Delta H^{\alpha }\times \frac{\partial \alpha }{\partial t}+wt_{BA}\times \Delta H^{\beta }\times \frac{\partial \beta }{\partial t}$$

where $wt_{Rubber}$ is the rubber mass fraction present in the elastomer compound, $wt_{BA}$ is the quantity of BA in mass fraction added to the rubber, ∂(α+β)/∂t is the coupled vulcanization-foaming reaction rate, ∂α/∂t is the rubber vulcanization reaction rate and ∂β/∂t is the BA reaction rate, $\Delta H^{\alpha }$ corresponds to the enthalpy value obtained for the rubber in Section 3.1, $\Delta H^{\beta }$ is the foaming enthalpy from Table 1 in Section 3.2 and $\Delta H^{\alpha +\beta }$ is the coupling enthalpy obtained in this Section.

In this compound, only a small percentage of BA is mixed with the rubber, approximately 1–3% in weight ($wt_{BA}$). Therefore, the rubber mass fraction in the elastomer compound is $wt_{Rubber}$ = 1 −$wt_{BA}$. When the BA is mixed with the rubber, an exothermic peak composed of the overlapping of two exothermic peaks can be observed in the green curve of the Figure 6 for the rubber+BA, one corresponding to the BA reaction at approximately 155–160 °C and the second one to the rubber vulcanization reaction at approximately 190–195 °C (see Figure 5). The second peak of the rubber+BA sample represented in the green curve in Figure 6 fits perfectly with the reaction of the rubber (blue curve) in the same figure. However, the first peak is completely different from the reaction of the isolated BA (compare the green curve with the yellow one in Figure 6). The BA decomposition reaction is produced at lower temperatures when it is mixed with the rubber, and its kinetic reaction rate is completely different from the kinetic observed in the decomposition reaction when it is alone. Therefore, it is assumed that the vulcanization process is not affected by the addition of BA and the vulcanization kinetic can be directly obtained from the experimental data presented in Section 3.1. Furthermore, to obtain the foaming rate (∂β/∂t), it is considered that the heat generated from the foaming reaction (called BAcalculated in Figure 6) is the difference of the heat produced in the coupled vulcanization and foaming reactions and the rubber vulcanization heat obtained at the same test conditions.

Once the data are in the format of foaming rate (∂β/∂t) vs temperature for each heating rate, the parameters of the kinetic reaction for the foaming mechanism in Equation (5) are obtained by solving a non-linear least-square problem.

### 3.4. Thermal Characterization

Elastomer thermal properties are characterized to model the heat transfer behaviour of the material. For an isotropic material, which is an affordable hypothesis for rubber behaviour, the heat transfer is defined by the specific heat and the conductivity of physical properties. The specific heat depends on the temperature and the vulcanization degree. However, the thermal conductivity only depends on the temperature. These dependences are introduced in the equations, which model the thermal elastomer behaviour.

#### 3.4.1. Specific Heat Capacity

The specific heat capacity ($C_{p}$) of the rubber is determined according to ISO 11357-4 standard. To compute the $C_{p}$ of the rubber, both uncured (non-vulcanized) and vulcanized rubber are analyzed in the temperature range where no transitions are observed; at phase transitions, part of the heat is consumed to produce a material state of higher energy, which is not used in raising the temperature and therefore a discontinuity in the heat capacity evolution is observed.

The samples for analyses are prepared by die-cutting 6 mm diameter discs from rubber sheets. For the test, sealed pans with a hole in the lid are used. First, to establish a base line, the program is carried out from 7 °C to 75 °C with an empty aluminum foil sample holder at the selected heating rate of 10 °C/min. This procedure is then repeated with a weighed sample added to the sample holder. In this case, both uncured and 100% vulcanized rubber samples are used to establish differences in their specific heat capacities. The same experiment is repeated for a reference material (pure α-alumina), which $C_{p}$ is known at different temperatures. As the sample is entirely enclosed by the sample holder and neither phase transitions nor sample mass changes are produced at the studied temperature heating range, the heat flow rate into the sample is given by Equation (7) [19]:

(7) $$\frac{\partial H}{\partial t}=m\times C_{p}\times \frac{\partial T}{\partial t}$$

where ∂H/∂t is the heat flow rate in J × s^−1^, *m* is the sample mass in grams, $C_{p}$ is the specific heat in J × g^−1^ × °C^−1^, and ∂T/∂t is the program heating rate in °C × s^−1^.

From DSC fundamentals, the definition of the $C_{p}$ and considering the specific heat capacity of the reference material ($C_{pr}$), the following relationship can be obtained:

(8) $$C_{p}=\frac{H}{h}\times \frac{m_{r}}{m_{s}}\times C_{pr}$$

where $C_{p}$ is the specific heat capacity of the sample, $C_{pr}$ is the specific heat capacity of the reference, $m_{s}$ is the sample weight, $m_{r}$ is the reference weight, *H* is the heat flow difference between the sample and the empty pan and *h* is the heat flow difference between the reference and the empty pan.

**Figure 7:**
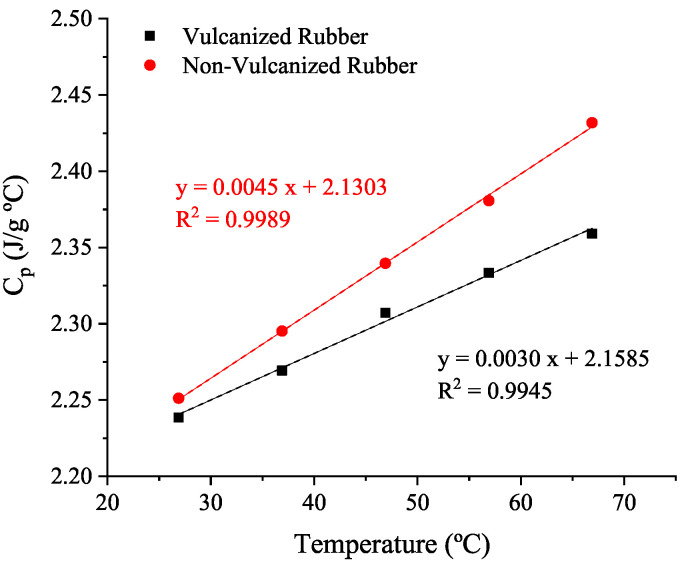
$C_{p}$ results for non-vulcanized and vulcanized rubber obtained at 10 °C/min heating rate from a known specific heat capacity of a reference material and the heat capacity of the empty DSC furnace.

The specific heat capacity, obtained for both uncured (non-vulcanized, $C_{p_{non-vulc}}$) and vulcanized rubber samples ($C_{p_{vulc}}$) by function of the temperature, is shown in Figure 7. The dependency of $C_{p}$ with the vulcanization degree ( $C_{p}^{\alpha }$) is included in the model by combination of two linear trends, using a mixing rule [20] as in Equation (9):

(9) $$\begin{array}{ccc} C_{p}^{\alpha } & = & \left(1-\alpha \right)\times C_{p_{non-vulc}}+\alpha \times C_{p_{vulc}}=\\ & & \left(1-\alpha \right)\times \left(a_{1}+b_{1}T\right)+\alpha \times \left(a_{2}+b_{2}T\right) \end{array}$$

where α is the vulcanization degree and parameters $a_{1}$, $b_{1}$, $a_{2}$ and $b_{2}$ corresponds with the linear regression obtained in Figure 7.

In the case of the foaming rubber, the dependency of the foaming degree in the specific heat capacity of the sample is computed taking into consideration the specific heat of the rubber at a certain vulcanization degree and the specific heat of the air enclosed in the elastomer compound from the bubbles generated, considered to be porosity in the mixing rule equation shown in Equation (10):

(10) $$C_{p}^{\beta }=\frac{C_{p}^{\alpha }\times \rho_{\alpha }\times \left(1-p\right)+C_{p}^{air}\times \rho_{air}\times p}{\rho_{\alpha }\times \left(1-p\right)+\rho_{air}\times p}$$

where β is the foaming degree, $\rho_{\alpha }$ and $\rho_{air}$ are the density of the rubber and air, respectively, in g × m^−3^, and *p* is the porosity level that is defined in Equation (11):

(11) $$p=1-\frac{1}{{\left(1+CFE\times \beta \right)}^{3}}$$

where CFE is a parameter that will be fitted in following sections and is related to the linear expansion that occurs when the foaming degree is unity (β=1).

#### 3.4.2. Thermal Conductivity

A DSC device allows the thermal conductivity of polymers to be rapidly determined typically with a measurement uncertainty of 10% according to the Mettler Toledo thermal analysis method [21], by measuring the melting behaviour of a pure metal placed on the top of a cylindrical sample or disc. DSC tests for measuring the thermal conductivity at the standard fusion temperature of indium and tin are performed using vulcanized rubber samples with different thickness (*h*) and the same area (A). Rubber samples are heated from 80 °C to 170 °C at a heating rate of 1 °C/min. Under stationary conditions, the heat flow, φ in W, through a body with a thermal resistance, $R_{s}$ in °C/W, is proportional to the temperature difference, ΔT in °C:

(12) $$\varphi =\frac{1}{R_{s}}\times \Delta T$$

The thermal resistance of the material is given by the material-dependent thermal conductivity and the geometry of the body:

(13) $$R_{s}=\frac{h}{\lambda A}$$

where λ is the thermal conductivity in W/m × °C, A the cross-sectional area in m^2^, of cylindrical samples with diameters *D* = 6 mm and *h* the length of the body in m.

Equation (14) can be easily obtained by combination of Equations (12) and (13), valid only during the melting process.

(14) $$\lambda =\frac{\varphi }{\Delta T}\frac{h}{A}$$

**Figure 8:**
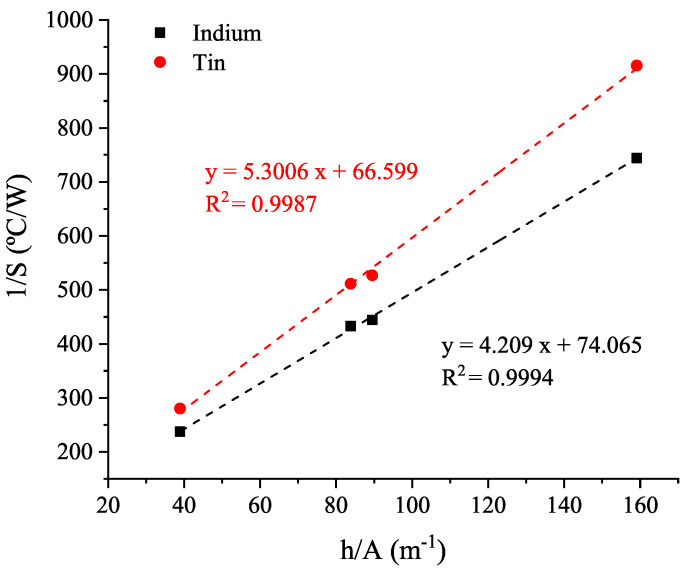
Representation of (1/Slope) vs. (thickness/Rubbersamplearea) to calculate the thermal conductivity obtained with each standard metal reference sample.

**Figure 9:**
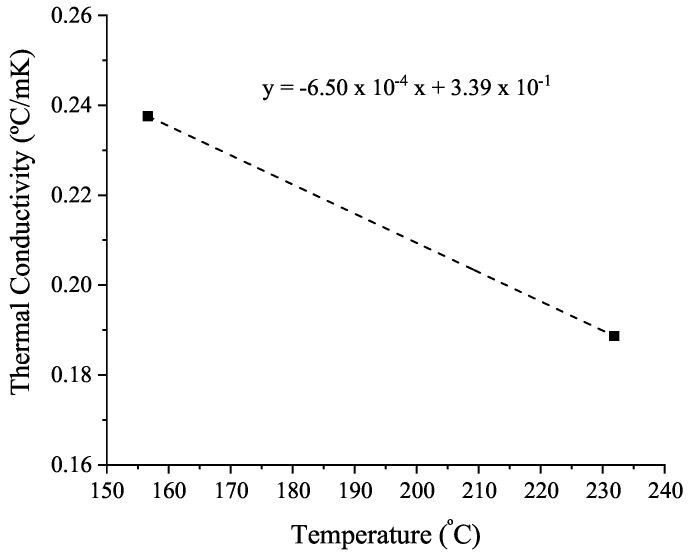
Thermal conductivity dependence with temperature.

The slope (*S* = φ/ΔT of the melting temperature peak) is measured from thermograms obtained with different rubber thickness samples. Considering then Equation (14), the thermal conductivity is determined from lineal regression of 1/S and h/A (Figure 8):

As two standard metal reference samples with different melting temperatures are used for the thermal conductivity determination, the dependence with temperature is obtained, as shown in Figure 9.

The thermal conductivity in the model is included for the rubber without foaming as:

(15) $$\lambda_{\alpha }=c_{1}+d_{1}T$$

where $c_{1}$ and $d_{1}$ are the parameters fitted in the linear regression in Figure 9.

According to Leach [22] and Bardy et al. [15], the thermal conductivity for rubbers with blowing agents, $\lambda_{\beta }$, can be obtained using a mixing rule as stated in Equation (16) as follows:

(16) $$\lambda_{\beta }=\lambda_{\alpha }\left(1-p\right)+\lambda_{air}p$$

where $\lambda_{\alpha }$ is the thermal conductivity of the rubber as described in Equation (15), $\lambda_{air}$ is the thermal conductivity of the air and *p* is the porosity level defined by Equation (11).

#### 3.4.3. Thermal Expansion of Rubber and Rubber+BA

The thermal expansion of the rubber sample is determined at different vulcanization temperatures by means of an internal procedure using a rheometer and a disc made of rubber of 40 mm diameter, which is die-cut from sheets of non-vulcanized rubber of approximately 1 mm thickness and placed between two metal plates. A pre-load of 0.2 N is established to maintain the upper plate in contact with the elastomer during the test, and so the rubber expansion can be determined. The rubber expansion is measured by the displacement of the rheometer upper plate, which corrects its instantaneous position in order to keep the pre-load on the sample when it is heated from 30 °C to 140, 150, 160, 180 and 200 °C at the heating rate of 20 °C/min. The temperature is controlled by means of two thermocouples, one placed in the rheometer furnace (without contact) and the second in contact with the upper plate. This way, both mechanisms causing the expansion of the rubber (convection and conduction heat transfer) can be considered in the material model.

**Figure 10:**
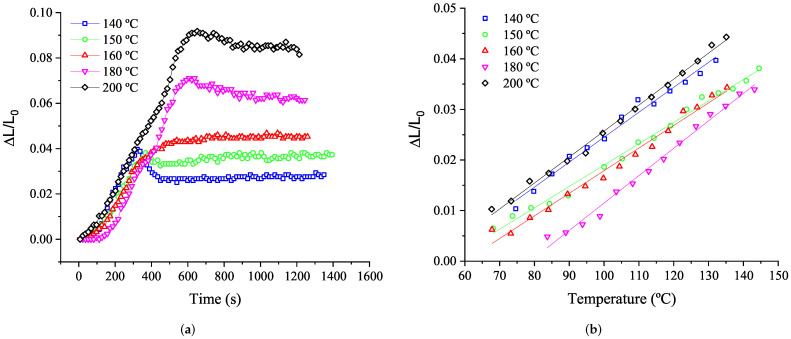
Rubber thickness evolution (rubber expansion) at 20 °C/min with vulcanization time (**a**) and temperature (**b**).

In Figure 10a, the rubber thickness increase with vulcanization time at different isothermal temperatures is presented. The rubber thickness increase due to the vulcanization expansion corresponds to the data achieved during the sample heating as, once the rubber is vulcanizing, the additional heat is employed in transforming the internal structure of the rubber due to the chemical reaction. Accordingly, Figure 10b shows the rubber thickness increase as a function of the temperature evolution. The slope of the expansion in the different tested rubber samples is quite similar at each test conducted, as can be seen in Figure 10b. Hence, it can be considered that the thermal expansion of the rubber (CTE) is approximately 5 × $10^{-4}$ m/m °C. Table 3 shows the slope and correlation factor obtained for the rubber expansion during the sample heating.

**Figure 11:**
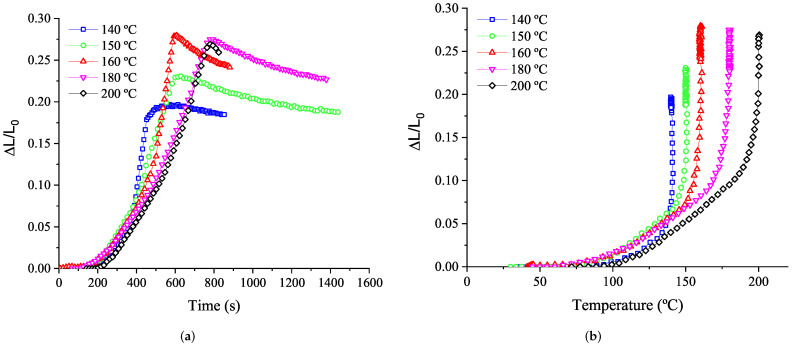
Rubber+BA expansion evolution at 20 °C/min with time (**a**). Foaming evolution: effect of isothermal temperature (**b**).

The same test conditions are performed for the rubber+BA. The resulting expansion (Figure 11) corresponds to the change in its volume due to the foaming process plus the thermal expansion of the rubber presented previously.

Foaming results of the rubber+BA compound in Figure 11 are used to determine the linear foaming expansion coefficient (CFE). Figure 11 shows the evolution of the thickness expansion with temperature and the maximal expansion obtained at different isotherms from 140 °C to 200 °C. The CFE can be obtained from the maximum expansion reached ($\Delta L/L_{0}$), assuming that the foaming reaction is totally completed, which means that the foaming degree is unity. Under this assumption, the CFE (dimensionless), is obtained according to Equation (17):

(17) $$CFE=max\left(\frac{\Delta L}{L_{0}}\right)-CTE\times \Delta T$$

where ΔL is the thickness increase referring to the initial thickness $L_{0}$ and ΔT the temperature increase.

### 3.5. Mechanical and Physical Characterization

Mechanical properties of the rubber and the rubber+BA at different vulcanization and foaming degrees are obtained at one isothermal temperature to establish a relationship between the vulcanization degree and the mechanical properties of both type of samples.

#### 3.5.1. Elastic Characterization of the Rubber and Rubber+BA Samples

The mechanical properties of the rubber and rubber+BA samples are determined in tensile mode from strip-size samples prepared by die-cutting from elastomer discs vulcanized in a Bohlin Gemini HRnano rheometer oven at 160 °C at certain vulcanization degrees. These samples are tested at laboratory temperature and humidity conditions of 23 °C and 50% of relative humidity and velocity of 10 mm/min. The material model for the stress analysis is assumed to be linear elastic. The behaviour of the vulcanized elastomer can be ideally assumed as hyperelastic; however, vulcanized elastomer compounds show the ability to undergo plastic deformation as described by Restrepo-Zapata [14]. In our case, a linear elastic behaviour is selected due to the fact that the strain level reached during the door seal profile extrusion process is low enough (below 10%) to maintain the quasi-linear behaviour before and after vulcanization.

**Figure 12:**
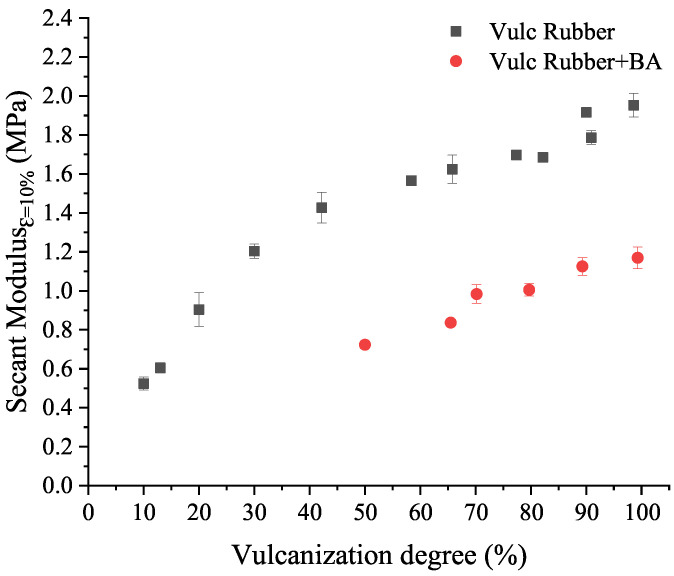
Comparison of secant modulus at 10% strain vs. vulcanization degree of rubber and rubber+BA samples vulcanized in a rheometer chamber at 160 °C.

The relationship between vulcanization and foaming degree and elastomer compound mechanical properties is established by means of the secant modulus calculated at 10% of strain (Figure 12). The evolution of the secant modulus at 10% strain in the rubber and rubber+BA samples, at different degrees of vulcanization, is similar. The secant modulus is approximately 40% lower in rubber+BA than in rubber samples, at vulcanization degrees from 80% to 100%. This reduction in tensile properties corresponds approximately with the value of the foaming area calculated from the rubber+BA vulcanized and foamed at 160 °C in the rheometer (see the next Section 3.5.2).

The dependence between the secant elastic modulus at 10% strain and the vulcanization degree is obtained from the rubber results shown in Figure 12. This relationship is defined through a 2nd-order polynomial equation as in Equation (18):

(18) $$E_{rubber}^{\varepsilon =10\%}=f\left(\alpha \right)=a\alpha^{2}+b\alpha +c$$

On the other hand, the dependence of this elastic modulus with the foaming degree is established based on the Mori–Tanaka (M-T) model particularized for spherical voids [23]. Under that assumption, the effective elastic modulus is defined by Equation (19):

(19) $$E_{Foamedrubber}^{\varepsilon =10\%}=\frac{9K_{\beta }G_{\beta }}{3K_{\beta }+G_{\beta }}$$

where $K_{\beta }$ is the bulk modulus of the foamed rubber and $G_{\beta }$ is the shear modulus of the foamed rubber, both in MPa, which are defined by Equations (20) and (21):

(20) $$K_{\beta }=\frac{E_{rubber}^{\varepsilon =10\%}}{3(1-2\nu )}\frac{2(1-p)(1-2\nu )}{2(1-2\nu )+p(1+\nu )}$$

(21) $$G_{\beta }=\frac{E_{rubber}^{\varepsilon =10\%}}{2(1+\nu )}\frac{\left(1-p)(7-5\nu \right)}{7-5\nu +8p-10\nu p}$$

where ν is the Poisson ratio (which is 0.45 for a quasi-incompressible material) and *p* is the porosity level defined by Equation (11).

#### 3.5.2. Structural Analysis of Rubber+BA

**Figure 13:**
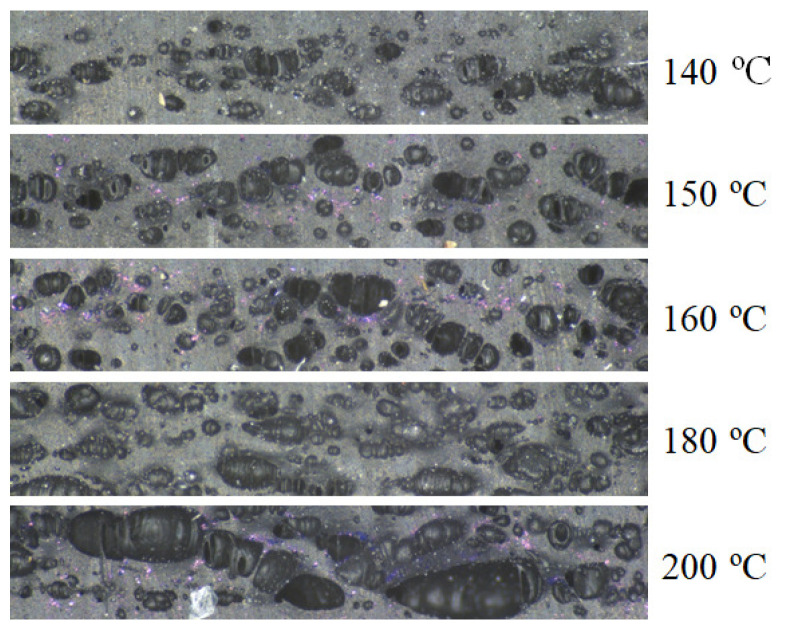
Rubber+BA samples area selection for the different isothermal temperatures under analysis.

**Figure 14:**
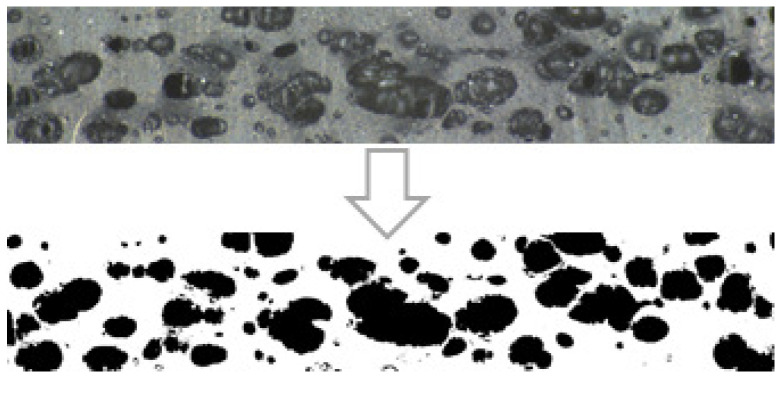
Image treatment for diameter and cell area calculation for the different isothermal temperatures under analysis.

Portions of rubber+BA samples foamed and vulcanized at different isotherms are characterized using a stereoscope, and the image analysis software ImageJ [24]. This information is used to calculate the evolution of cell area as a function of an isothermal temperature and the evolution of the bubble diameter (Figure 13 and Figure 14). Six areas of each sample foamed and vulcanized at different isotherms are analyzed to compute the total cell area (a.k.a. cell area), as a ratio of a total area of cells in a specific area of the rubber compound. The study area for each one has the same size, equal to 32.28 mm^2^. The percentage of cell area for each of the areas is calculated by means of Equation (22).

(22) $$Cell\;area=\frac{Black\;area}{Total\;area}$$

**Figure 15:**
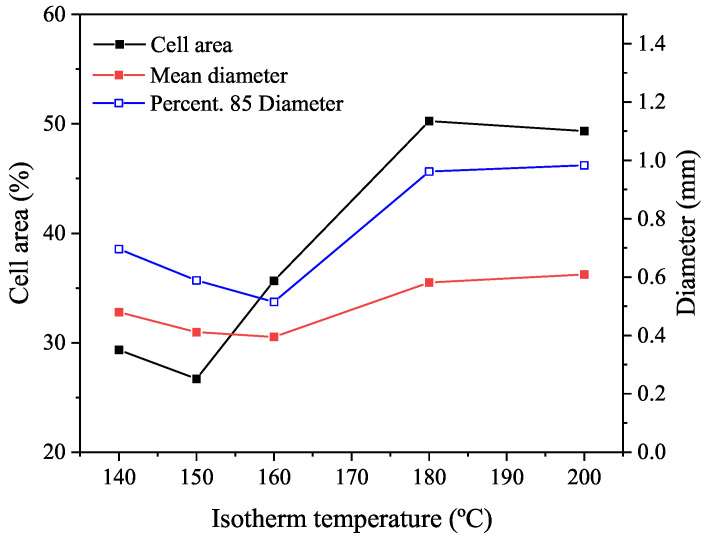
Evolution of cell area as a function of isothermal temperature.

Figure 15 presents the results for three different parameters—cell area, average cell diameter and 85th percentile-diameter-value—at each foaming and vulcanization temperature. There is an increase of cell area and diameter as the isothermal temperature increases, until the maximum cell area of 50%. In the range approximately from 160 °C to 180 °C, the increment of area seems to be faster than at the other lower isothermal temperatures. The observed tendency of decreasing the diameter when the isothermal temperature increases from 140 °C to 160 °C suggests that the rubber vulcanization and BA decomposition kinetics show similar velocity; however, when the isothermal temperature increases from 160 °C to 180 °C, the BA decomposition kinetic seems to be dominant.

Cell area and diameter evolution with processing temperature shows the same tendency to the one observed for the rubber+BA expansion (Figure 11), i.e., a pronounced increase in the cell area, cell diameter and rubber+BA expansion is observed from 160 °C to 180 °C, following by a gradual decrease from 180 °C to 200 °C, probably due to the fact that the bubbles tend to collapse. Therefore, the consistency of results can be confirmed.

### 3.6. Material Model Coupling

The previous characterization is used to develop a numerical material model able to reproduce the vulcanization and foaming behaviour of the rubber aimed for use in FE codes. As demonstrated by experimental evidence in former sections, the model must include the coupling of the different fields: chemical (vulcanization and foaming kinetics), thermal and mechanical.

The proposed model includes the equations that are defined in previous sections for the individual behaviour of each material property. However, the interaction between the vulcanization and foaming reactions themselves and with other elastomer properties must be also taken into account, as explained in Section 1.3 and Section 3.3.

The foaming process is, essentially, a bubble growth process. This bubble growth mechanism has been introduced in the literature by several authors [10,11,12,13], its fundamental principle being coupled mass and momentum conservation. In the literature, it is normally defined in terms of viscosity, surface tension and pressure inside the bubble [13]. However, this paper approaches the foaming mechanism in a different way, since most FE codes use the displacement variable as degree of freedom (DoF) instead of velocity. For that reason, the foaming mechanism cannot be implemented as a function of viscosity, which is a velocity-dependent property, but as function of a displacement-dependent property related to the elasticity of the elastomer. In this work, the elasticity property chosen to establish this relationship is the elastic modulus. Previously, it was noticed that the secant elastic modulus at 10% strain is related to the vulcanization degree (Figure 12). Therefore, the foaming mechanism dependence with vulcanization degree is introduced considering that bubble growth depends on viscosity through the elastic modulus of the material. As the vulcanization reaction progresses, the viscosity of the elastomer increases and so its elastic modulus does. Hence, we propose to introduce the dependency between the foaming and vulcanization degrees using a function g(α) inside Equation (5), which allows the consideration of the dependence of the vulcanization and foaming degree with the elasticity (elastic modulus) of the elastomer. This function is proposed as stated by Equation (23):

(23) $$g\left(\alpha \right)=e^{-\alpha h}$$

where *h* is a parameter calculated from the fitting of an exponential equation for the data in Figure 12, expressed as $\alpha \;vs\;\hat{1/\hat{E}}$.

This model, called from this point onwards the initial model, is proved for the test of rubber+BA in Section 3.4.3 (Figure 11). A comparison is made in terms of strain, ϵ, or equivalent elastomer thickness expansion, $\Delta L/L_{0}$, which depends on the rubber thermal expansion and the thickness increase produced due to the foaming reaction. Then, the strain can be computed through Equation (24).

(24) ϵ(β;ΔT)=β×CFE+ΔT×CTE

**Figure 16:**
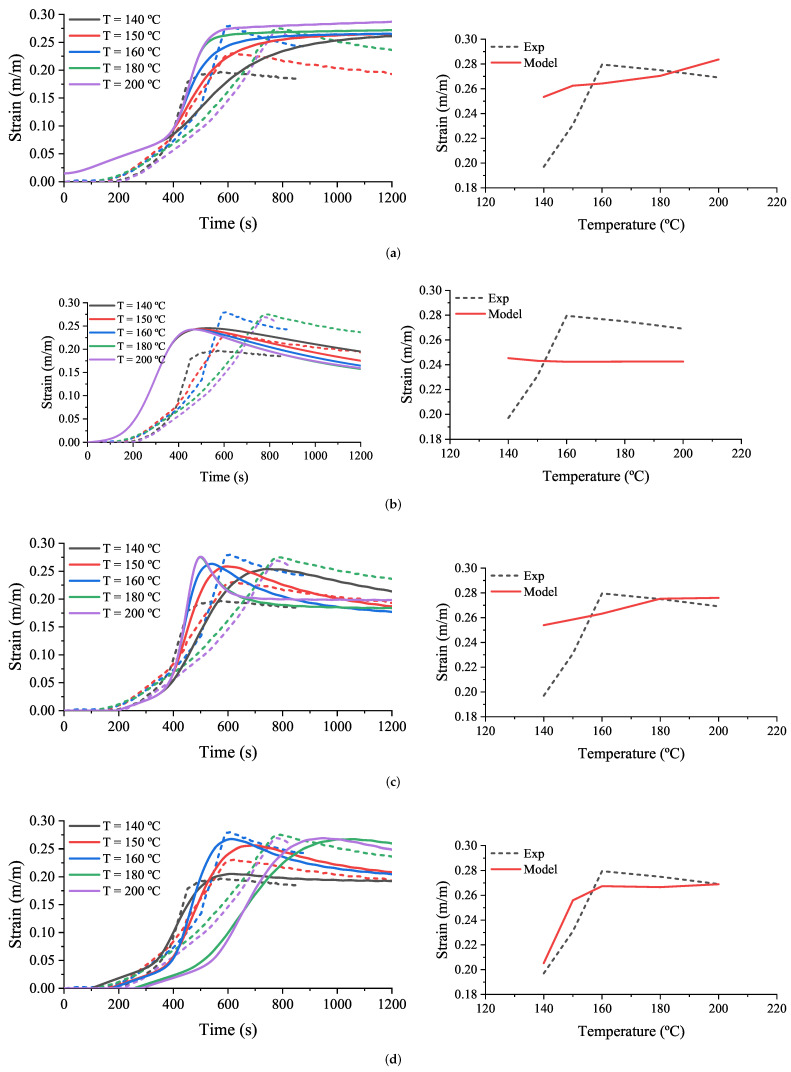
Comparison between different material models (solid lines) and experimental characterization results (dash lines). (**a**) Initial model; (**b**) Wang model; (**c**) Wang-Beta model; (**d**) Wang-Beta model-E variable.

The results from the comparison between the model and test results are shown in Figure 16a. The parameters used for this model are summarized in Table 4 (the authors remark that $Q_{total}^{\beta }$ is expressed as a function of the instantaneous density of the rubber+BA, $\rho_{\beta }$).

As can be concluded from Figure 16a, this model is able to reproduce neither the measured behaviour nor the trends of the strain overtime or the maximum strain reached with the maximum temperature.

Due to the observed mismatch, a different approach is proved for modelling the foaming mechanism in the rubber+BA. For this purpose, the Equation (5) is replaced by an approach given by Wang et al. [13] with minor changes. The amount of gas evolved is expressed with Equation (25) and the amount of bubble volume is expressed according to Equation (26).

(25) $$\frac{\partial N}{\partial t}=K_{0}\;e^{-E/RT}\left(N_{0}-N\right)$$

where *N* is the quantity of BA in moles of the total amount introduced that in fact decomposes or reacts, $N_{0}$ is the initial amount of BA, and $K_{0}$ and *E* are parameters of the Arrhenius-type equation.

(26) $$\frac{\partial V}{\partial t}=\frac{3NRT}{4\mu }-\frac{3P_{\infty }}{4\mu }V$$

where *V* is the volume of bubbles in m^3^, μ is the viscosity in N × s/m^2^ and $P_{\infty }$ is the pressure far from the bubble in Pascals. Both the rubber viscosity and the bubble pressure depend on the vulcanization degree, and they are considered in the model by means of two linear regression Equations (27) and (28).

(27) $$\mu =\mu_{uncured}+\alpha \times f_{mu}$$

where $\mu_{uncured}$ is the value that takes μ when the rubber is uncured, and $f_{mu}$ is the slope of the linear regression.

(28) $$P_{\infty }=P_{\infty }^{uncured}+\alpha \times f_{P}$$

where $P_{\infty }^{uncured}$ is the value that takes $P_{\infty }$ when the rubber is uncured and $f_{P}$ is the slope of the linear regression.

In this case, the strain is calculated according to Equation (29).

(29) $$\epsilon ={\left(1+V\right)}^{1/3}-1$$

The model is implemented using Jupyter notebooks to analyze the effects of some parameters such as $N_{0}$, $\mu_{uncured}$, $P_{\infty }^{uncured}$, $f_{mu}$, $f_{P}$, $K_{0}$ and *E*. There are multiple combinations of parameters available to capture the experimental behaviour. One of the best fits is shown in Figure 16b. The ‘relaxing’ effect observed in the test can be introduced by means of Equations (27) and (28); however, the Wang model is not able to capture the behaviour observed experimentally.

Then, a combination of both approaches, called the Wang–Beta model, is implemented. The foaming law is obtained using Equation (5) without the function g(α) and the volume of bubbles with Equation (26), with $N=\beta \times N_{0}$. Parameters for the best fit are searched for with Jupyter notebooks, resulting in the one shown in Figure 16c. Although this model reproduces a global trend, the maximal strain rate observed in the experimental test is reached at a lower temperature in comparison with the model in which it is reached at higher temperature. Additionally, the maximum strain predicted by the model for the case of 140 °C is significantly higher than the one expected according to the experimental measurements. Both facts leave room for model improvement.

For this purpose, a new model based on the Wang–Beta model with a change in the value of the parameter *E* of term $K_{2}$ in Equations (2) and (5) is implemented to prove its predictive goodness. Hence, the values stated for the Wang–Beta model with the variable of the parameter *E* are compiled in Table 5 and results are depicted in Figure 16d. The best approximation to the experimental results are obtained considering the bubble volume and the amount of gas evolved in the foaming reaction in combination with Equation (2) for the foaming kinetic and fitting parameter E in the best way, changing the term $K_{2}$ in Equations (2) and (5).

The Wang–Beta model with modified E-parameter significantly improves the prediction of the experimental measurements of the thickness expansion ($\Delta L/L_{0}$, also referred as “strain” in Figure 16) since it is able to catch main tendencies as well as the dependency between the maximum temperature and strain. It must be highlighted that this rheometer experiment is a specific case in which the heating mechanism is generated mainly by conductive heat transference, while the model was fitted with experimental data from a DSC experiment, where the heating mechanism is mainly governed by convective heat transference. The different heating mechanisms in the rheometer and the DSC device can impact the accuracy of the developed model, but does not make invalid its prediction capability, which in fact is very significant.

Moreover, the material model is defined from an analytical model, which considers material behaviour to be isotropic, i.e., the model does not take into account any specific dimension, just one, while conducted experiments are three-dimensional, since properties in real samples are measured. This fact, far from being a drawback, makes the developed model even more powerful for its practical application, since, despite being a source of inaccuracy, it seems not to be so outstanding when observing the obtained results.

## 4. Discussion

The DSC results explained in Section 3 reveal on one hand that the vulcanization and foaming reactions are produced within the same range of temperatures and, on the other hand, how the interaction of both reactions produces a change in the BA decomposition kinetic behaviour when mixed with the rubber. In this particular case of rubber+BA material, the vulcanization kinetic reaction remains equal, comparing the kinetic of the rubber vulcanization. However, the foaming kinetic reaches its maximum velocity at lower temperatures, when it is compared with the BA alone, at the same heating conditions. Consequently, the foaming rate (∂β/∂t) is calculated as the difference between the heat produced during the coupled vulcanization-foaming reaction and the rubber vulcanization heat, considering the percentage of BA added to the rubber+BA compound. This behaviour is also reported in other published works. Restrepo et al. [2,14] realized that when the BA (OBSH) is mixed with the EPDM (with a sulphur-based curing system), it could be observed that the BA reaction decreases its velocity almost twice as slowly as the BA alone. Additionally, the maximum rate of the coupled vulcanization-foaming kinetic reactions takes place at lower temperatures in comparison with isolated BA decomposition reaction. Restrepo et al. concluded that the BA decomposition reaction is affected by the additives of the elastomer matrix. However, the opposite effect is observed by Vahidifar et al. [4] in a polyisoprene rubber (IR) foam with azodicarbonamide (ADC) as chemical BA and zinc oxide (ZnO) as vulcanization activator. Curing rates of the IR foam compound without ADC are faster than with ADC; i.e., the IR shows a shorter curing time when ADC is absent. They relate this observation to three possible causes. First, the bubbles generated in the compound might be acting as heat insulators decreasing the reaction rate. Second, there is the possible competition of the ZnO activator between both reactions, which might lead to a partial participation in each reaction, resulting in a faster decrease of ZnO efficiency on the rubber curing. Third, ADC decomposition products might have some impact on the IR curing. In our case, the elastomer compound composition is unknown, but it is probable that the rubber blend contains one or more chemicals that could influence the BA decomposition kinetic reaction in combination with the EPDM rubber matrix. A structural analysis of portions of foamed rubber+BA samples at different foaming and vulcanization temperatures reveals that cell diameters are practically constant from 140 °C to 160 °C with a small increase in cell area. This fact suggests that the rubber vulcanization and BA decomposition reactions are produced simultaneously with similar velocity in the analyzed temperature range. Then, a significant increase in both parameters from 160 °C to 180 °C is produced, which corresponds to a faster velocity of the BA decomposition reaction. From 180 °C to 200 °C, a slight decrease in area and cell diameters is observed, probably due to bubble collapse. Similar results have been found by other authors in their investigations [3,4,5].

Moreover, a new experimental rheometer test is developed in this work to determine the CTE of the rubber and the CFE of the rubber+BA. Furthermore, the experimental results of this test, made on rubber+BA, has been used to calibrate several forms of a constitutive numerical model for its implementation in FE codes, by using the material model equations established from the global experimental strategy (kinetics, thermal, mechanical and physical characterization of rubber and rubber+BA samples) and phenomenological physical laws. The numerical material model reproduces with enough accuracy the experimental cellular rubber expansion recorded during the rheometer tests and achieves final expansion values at each isothermal temperature with a precision of 90–99%. Finally, the cellular rubber expansion values follow the same trend as the one observed from the structural analysis of vulcanized and foamed samples at different isothermal temperatures. This analysis reveals an increase in cell area up to 50%, which perfectly fits the observed proportional loss of mechanical properties in the function of the vulcanization and foaming degree. These results are consistent with those obtained by Zakaria et al. in EPDM foams [5].

## 5. Conclusions

The experimental methodology developed for the characterization of the vulcanization and foaming processes of an EPDM cellular rubber allows the identification and quantification of the interaction between vulcanization and foaming reactions. Additionally, the thermal properties, cell diameter and area and mechanical properties are obtained and their relationship, with the vulcanization and foaming reaction kinetics, established.

This novel experimental methodology allows the definition of the relationship established between the coupling of chemical reactions (vulcanization and foaming kinetics) and thermal and mechanical properties of the elastomer compound, and provides valuable information to generate a numerical material model able to reproduce with reasonable accuracy the experimental evidence explained in Section 3.4.3, and specifically depicted in Figure 11 and Figure 16d.

Hence, the experimental methodology described in this paper allows the obtaining of sufficient parameters to define material models able to reproduce with enough accuracy the final properties of components made of foamed rubber compounds, through the performance of short and relatively simple laboratory tests, in which small amounts of rubber samples that are easy to achieve are used.

The implemented material model can be used to simulate manufacturing processes in which rubber+BA is involved. It can be used to improve and reduce manufacturing defects produced in industrial applications, such as, for instance, in automotive sealing profile production. In this kind of industrial application, final profile properties and the profile shape itself depend on molecular changes during the vulcanization process and they are normally fitted by a trial-and-error process. For that reason, the material model is used for modelling a real-time extrusion line of this automotive component by Viejo [25] and the resulting data, obtained with the finite-element (FE) simulation model, are used to build a Reduced-Order Model (ROM). The results of the study allow the identification of manufacturing uncertainties associated not only with different material characterization and process parameters but also with aleatory ones as well as those of epistemic origin. This information can be very useful for appropriately handling uncertainty propagation across manufacturing lines, and opens a research window into the development of in-line sensors. These sensors could monitor the evolution of one or more product properties during the manufacturing process. By introducing the information obtained from them in the material model, it could be possible, automatically acting on the most influential control parameters of the process, to minimize product rejections. Thus, the material model would be continuously re-fed and it would establish the necessary changes in the production line instantly, eliminating the human factor and reducing reaction times and productivity losses. The final conclusion is that the production of industrial automotive sealing profiles, manufacturing defects, and production costs can be reduced by increasing the knowledge of the kinetics reaction evolution and physical and mechanical changes along extrusion lines.

## Figures and Tables

**Table 1:** Dynamic DSC results of blowing agent (BA).

Heating Rate	Enthalpy Second Peak	T Peak
(°C/min)	(J/g)	(°C)
2.5	494.72	163.44
5	506.56	168.58
7	492.93	171.83
10	432.96	173.13

**Table 2:** Dynamic DSC results of rubber+BA samples.

Heating Rate	Enthalpy	T Peak
(°C/min)	(J/g)	(°C)
5	11.80	157.40
7	11.74	158.40
10	12.01	158.70
20	9.90	164.40

**Table 3:** Linear regression equations and correlation values of $\Delta L/L_{0}$ vs. heating temperature at 20 °C/min.

T (°C)	Slope	$R^{2}$
140	$4.93\times 10^{-4}$	0.984
150	$4.23\times 10^{-4}$	0.993
160	$4.47\times 10^{-4}$	0.985
180	$5.41\times 10^{-4}$	0.990
200	$5.13\times 10^{-4}$	0.996

**Table 4:** Summary of the model: equations, parameters and values.

Quantity (Symbol)	Equation	Fitted Value
Density ($\rho_{\alpha }$)	—	$1.1\times 10^{6}$ g/m^3^
Specific heat capacity ($C_{p}$)	(9)	$a_{1}=2.1303\times 10^{0}$
		$b_{1}=4.4663\times 10^{-3}$
		$a_{2}=2.1585\times 10^{0}$
		$b_{2}=3.05\times 10^{-3}$
Thermal conductivity (λ)	(15)	$c_{1}=3.393682\times 10^{-1}$
		$d_{1}=6.4995\times 10^{-4}$
Coefficient of thermal expansion (CTE)	—	$5\times 10^{-4}$ m/m °C
Coefficient of foaming expansion (CFE)	—	$2.3\times 10^{-1}$
Vulcanization degree (α)	(1)	$Q_{total}^{\alpha }=2.2638\times 10^{6}$ W/m^3^
	(2)	$A1=1.24284\times 10^{-6}$
	(3)	$E1=2.9768\times 10^{4}$
		$A2=6.37949\times 10^{7}$
		$E2=8.4506\times 10^{4}$
		$B_{\alpha }=1.01613\times 10^{0}$
		$m=3.64951\times 10^{-1}$
		$n=1.12585\times 10^{0}$
Foaming degree (β)	(4)	$Q_{total}^{\beta }$ = 7200 $\times \rho_{\beta }$ W/m^3^
	(5)	$A1=5.23315\times 10^{3}$
	(3)	$E1=7.1926\times 10^{4}$
		$A2=7.22514\times 10^{6}$
		$E2=7.0500\times 10^{4}$
		$B_{\beta }=1.0000\times 10^{0}$
		$m=6.05424\times 10^{-1}$
		$n=8.19961\times 10^{-1}$
	(23)	$h=5.2265\times 10^{0}$
Elastic modulus (*E*)	(18)	$a=-1.58120\times 10^{0}$
		$b=3.13977\times 10^{0}$
		$c=2.9015\times 10^{-1}$

**Table 5:** Values used in the fitting of the model.

T	E for $K_{2}$ in	E for $K_{2}$ in
(°C)	Equation (2)	Equation (5)
140	78,081	69,093
150	83,810	71,629
160	84,475	71,912
180	91,626	78,680
200	93,787	80,933

## Data Availability

Not applicable.

## Abbreviations

The following abbreviations are used in this manuscript:

EPDM	Ethylene Propylene Diene
FE	Finite Element
PU	Polyurethane
EVA	Ethylene Vinyl Acetate
NR	Natural Rubber
SBR	Styrene-Butadiene Rubber
CR	Chloroprene Rubber
ACM	Alkyl Acrylate Copolymer
NBR	Acrylonitrile Butadiene Rubber
TSH	Toluene Sulphonyl Hydrazide
OBSH	4,4′- Oxybis(Benzene Sulphonyl Hydrazide)
DSC	Differential Scanning Calorimetry
TGA	Thermogravimetric Analysis
Rubber	Rubber matrix without blowing agent
BA	Blowing Agent
Rubber+BA	Rubber with blowing agent
CTE	Thermal Expansion Coefficient
CFE	Foaming Expansion Coefficient
DoF	Degree of Freedom
IR	Polyisoprene rubber
ADC	Azodicarbonamide
ZnO	Zinc oxide
